# Sustainable Weed Management: The Effects of Applying Pre- and Post-Emergence Herbicides to *Medicago ruthenica*

**DOI:** 10.3390/plants14060864

**Published:** 2025-03-10

**Authors:** Qiang Li, Zhongwei Ren, Hui Xu, Wenying Wang, Yarong Zhang, Fan Huang, Linqing Yu, Jun Li

**Affiliations:** 1 Key Laboratory of Herbage & Endemic Crop Biology, Ministry of Education, School of Life Sciences, Inner Mongolia University, Hohhot 010020, China; liqiang@emails.imau.edu.cn (Q.L.); rzw1696840@163.com (Z.R.); xh15201297391@163.com (H.X.); wywang@imu.edu.cn (W.W.); linqing_yu@126.com (L.Y.); 2 College of Agriculture, Inner Mongolia Agricultural University, Hohhot 010019, China; 3 Inner Mongolia Pratacultural Technology Innovation Center Co., Ltd., Hohhot 010070, China; cczxzyr123@163.com; 4 Grassland Research Institute, Chinese Academy of Agriculture Sciences, Hohhot 010010, China; huangfan@caas.cn

**Keywords:** weed management, herbicides, *Medicago ruthenica*, sustainable agriculture

## Abstract

*Medicago ruthenica* is a forage legume crop that is widely used as fodder and for ecological restoration in arid and semi-arid areas in Northcentral Asia. During the seedling stage, weeds challenge the growth and development of *M. ruthenica*, especially in fields sown for seed production. However, strategies to effectively control weeds in crops of *M. ruthenica* using herbicides have not been investigated. We evaluate the efficacy of different herbicides that control pre- and post-emergence of weeds in *M. ruthenica*. The results indicated that the most effective pre-emergence herbicides, imazethapyr (1530 mL ha^−1^) and flumetsulam (120 mL ha^−1^), resulted in crop safety and soil microbial community equivalent to a weed-free check. The most effective post-emergence herbicides are imazethapyr + haloxyfop-P (1800 + 600 mL ha^−1^) and 2,4-DB + haloxyfop-P (2250 + 600 mL ha^−1^). These herbicide treatments demonstrate effective control of most weeds (*A. retroflexus, C. album,* and grasses) while ensuring crop safety. Application of these herbicides to control weeds in *M. ruthenica* prior to or after their emergence represents a viable strategy for their control and also improve agricultural viability and crop yield and quality. Our research contributes to sustainable agriculture and ecological restoration in arid regions.

## 1. Introduction

*Medicago ruthenica,* a diploid (2n = 2x = 16) perennial legume [1], is an important forage species that is widely distributed in areas of northern China, Siberia, and Mongolia [2,3]. *M. ruthenica* is a perennial forage species in natural grasslands and is also used for ecological modification because of its tolerance to cold and drought stress, especially on the Mongolian plateau [4,5]. Continued demand for increased livestock production and environmental protection in China has increased the demand for commercial *M. ruthenica* seed. However, seed production in the field is significantly restricted by weed competition [6] (about 70% to 80% decrease in *M. ruthenica*), and also, there is a high cost to purify seed due to weed seed contamination [6]. Weeds competition is a major limiting factor for seed production of *M. ruthenica*.

Weeds are a global problem that influences crop growth and development [7]. With the increased mechanization of modern agriculture production, controlling weeds is also becoming increasingly important [7]. The use of herbicides to control weeds is one strategy [8] that is more efficient, reliable, and cost-effective than traditional manual and mechanical weeding [9]. Herbicides are typically applied to act on weeds prior to or following their emergence [10]. The application of pre-emergence herbicides to soil reduces weed populations by inhibiting seed germination for 0–30 d [11]. Post-emergence herbicides are applied to secondary weed infestations at the 2–4 leaf stages [12]. To obtain desired results, accurate weed identification and an understanding of their life histories are essential [13]. Effective control is best achieved through the application of an appropriate herbicide at an appropriate time and dosage, using an appropriate application method [14]. Because legume forage develops slowly after sowing, weed grasses and broadleaf weeds can outcompete it [15]. This reduces legume yield and quality [16], mainly through failed crop establishment, reduced production, and increased costs associated with harvesting and purifying [17,18]. For example, weed infestation can reduce alfalfa (*Medicago sativa*) seed yield by 34–48% [19] and decrease forage yield by about 45% [20]. Pre- and post-emergent herbicidal control of weeds can positively affect legume crops [21]. Saflufenacil temporarily injured broadleaf plantain (*Plantago major*) and ribwort plantain (*Plantago lanceolata*) and had few negative impacts on alfalfa in the field [22]. The use of imazethapyr as a pre- and post-emergent herbicide effectively controlled weeds and improved the yield of Egyptian clover (*Trifolium alexandrinum*) [23]. The efficacy of and degree to which pre- and post-emergence herbicides injure legume crop species varies greatly [21].

Compared with alfalfa, *M. ruthenica* is slower growing, indicating that weeds can outcompete it for soil water, nutrients, or light resources, especially during early growth stages (0–60 days). This also poses challenges for seed field establishment. However, no information is available on chemical weed control in *M. ruthenica*. In this study, we evaluated the selectivity and sensitivity of pre- and post-emergent herbicides in controlling weeds in *M. ruthenica* experimental fields. To our knowledge, this is the first study on chemical weed selectivity and sensitivity for *M. ruthenica*.

## 2. Results

### 2.1. Weed Species and Their Density

Prevalent annual broadleaf and grass weed species in *M. ruthenica* control plots are detailed in Supplementary Table S1. Seven annual weed species in five families occurred in experimental plots over the two years (Supplementary Table S1); most were annual broad-leafed species, of which *Chenopodium album* (40.64–44.60%) and *Amaranthus retroflexus* (27.23–28.40%) dominated.

### 2.2. Pre-Emergence Herbicide Weed Control Efficiency

**Figure 1:**
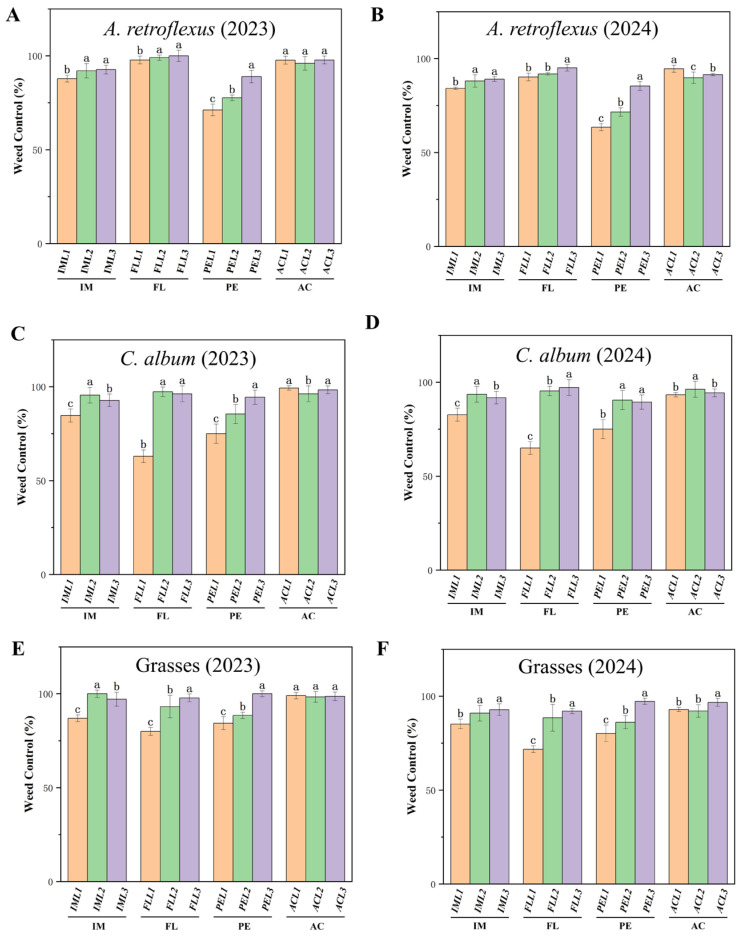
Effect of pre-emergence herbicides on weed control (%) in different treatments at 30 DAS for *A. retroflexus* in (**A**) 2023 and (**B**) 2024; *C. album* in (**C**) 2023 and (**D**) 2024; and grasses in (**E**) 2023 and 2024 (**F**). Pre-emergence herbicides imazethapyr (IM, IML1, IML2 and IML3 denote different concentrations), flumetsulam (FL, FLL1, FLL2, FLL3 denote different concentrations), acetochlor (AC, ACL1, ACL2, ACL3 denote different concentrations), and pendimethalin (PE, PEL1, PEL2, PEL3 denote different concentrations). All treatments were measured with three replications. Different letters indicate significant differences between treatments (Duncan test at *p* < 0.05).

All pre-emergence herbicides significantly reduced weed density compared with the weed control (Figure 1). For example, in IML1-treated plots, control of *A. retroflexus* (Figure 1A,B) and *C. album* (Figure 1C,D) had a control efficiency of over 82%, and that of grass had a control efficiency of 85% (Figure 1E,F). In IML2 and IML3-treated plots, control of *A. retroflexus* (Figure 1A,B), *C. album* (Figure 1C,D), and grass (Figure 1E,F) had a high efficacy (>90%). In FLL1 treatments, control of *A. retroflexus* (Figure 1A,B) was 90%, and that for *C. album* (Figure 1C,D) and grass (Figure 1E,F) was 62–79%. For FLL2 and FLL3 treatments, these weed species showed high efficacy (>90%). All applications of acetochlor were highly effective at controlling weeds (>90%).

### 2.3. Post-Emergence Herbicide Weed Control Efficiency

**Figure 2:**
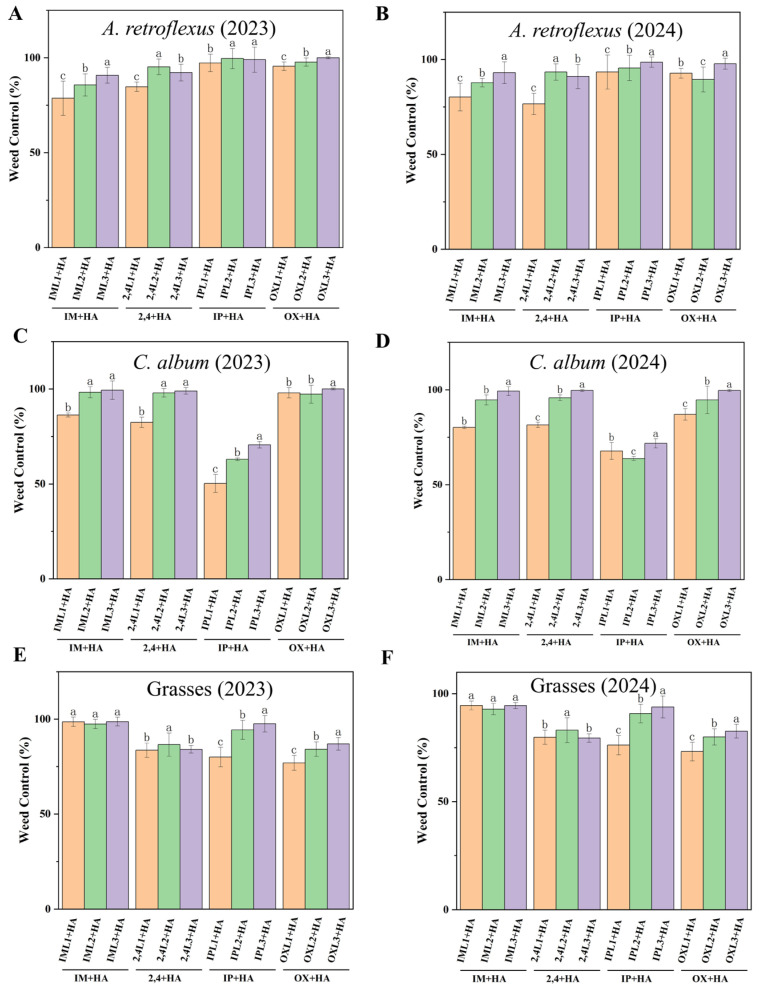
Effect of post-emergence herbicides on weed control (%) in different treatments at 60 DAS for *A. retroflexus* in (**A**) 2023 and (**B**) 2024; *C. album* in (**C**) 2023 and (**D**) 2024; and grasses in (**E**) 2023 and (**F**) 2024. Post-emergence herbicides of imazethapyr + haloxyfop-P (IM + HA, IML1 + HA, IML2 + HA, IML3 + HA denote different concentrations), imazapic + haloxyfop-P (IP + HA, IPL1 + HA, IPL2 + HA, IPL3 + HA denote different concentrations), 2,4-DB + haloxyfop-P (2,4 + HA, 2,4L1 + HA, 2,4L2 + HA, 2,4L3 + HA denote different concentrations), and oxyfluorfen + haloxyfop-P (OX + HA, OXL1 + HA, OXL2 + HA, OXL3 + HA denote different concentrations). All treatments were measured with three replications. Different letters indicate significant differences between treatments (Duncan test at *p* < 0.05).

Haloxyfop-P was tank-mixed with herbicides to evaluate weed control efficiency. At 60 DAS, weed densities were substantially reduced in all post-emergence herbicide treatments. Each of 2,4L2 + HA, 2,4L3 + HA, OXL1 + HA, OXL2 + HA, and OXL3 + HA effectively controlled (>89%) *A. retroflexus* (Figure 2A,B). Excellent control of *C. album* (Figure 2C,D) was achieved in IM + HA, 2,4 + HA, and OX + HA treatments (>80%). Each of the IM + HA, 2,4 + HA, and IP + HA treatments controlled grasses (Figure 2E,F) with a control efficiency of over 76%.

### 2.4. Effects of Herbicides on M. ruthenica Growth and Injury

The toxicity of different herbicide treatments was evaluated in *M. ruthenica*. At 30 DAS, different levels of injury occurred following the application of each pre-emergence herbicide (Table 1). Crop injury was consistently 1 in IML2 and FLL3 treatments. Compared with weed-free control, *M. ruthenica* dry matter and plant height were not significantly reduced in IML2 and FLL3 treatments. Crop injuries in acetochlor and pendimethalin treats were rated 4, respectively, with both plant height and dry matter significantly inhibited. Post-emergence herbicide crop injuries are detailed in Table 2. Injury from imazethapyr + haloxyfop-P tank mix (IML1 + HA, IML2 + HA, IML3 + HA) rated 1, as did (excepting the 2,4L3 + HA treatment) application of 2,4-DB + haloxyfop-P tank mix (2,4L1 + HA, 2,4L2 + HA). Conversely, imazapic and haloxyfop-P tank mix (IPL1 + HA, IPL2 + HA, IPL3 + HA) and oxyfluorfen and haloxyfop-P tank mix (OXL1 + HA, OXL2 + HA, OXL3 + HA) had crop injury ratings of 3 or 4. Application of different post-emergence herbicide combinations affected plant height and dry matter. Compared with the weed-free samples, *M. ruthenica* dry matter and plant height were not significantly reduced in IML3 + HA and 2,4L2 + HA treatments.

### 2.5. Effects of Pre-Emergence Herbicides on Soil Microorganisms

**Figure 3:**
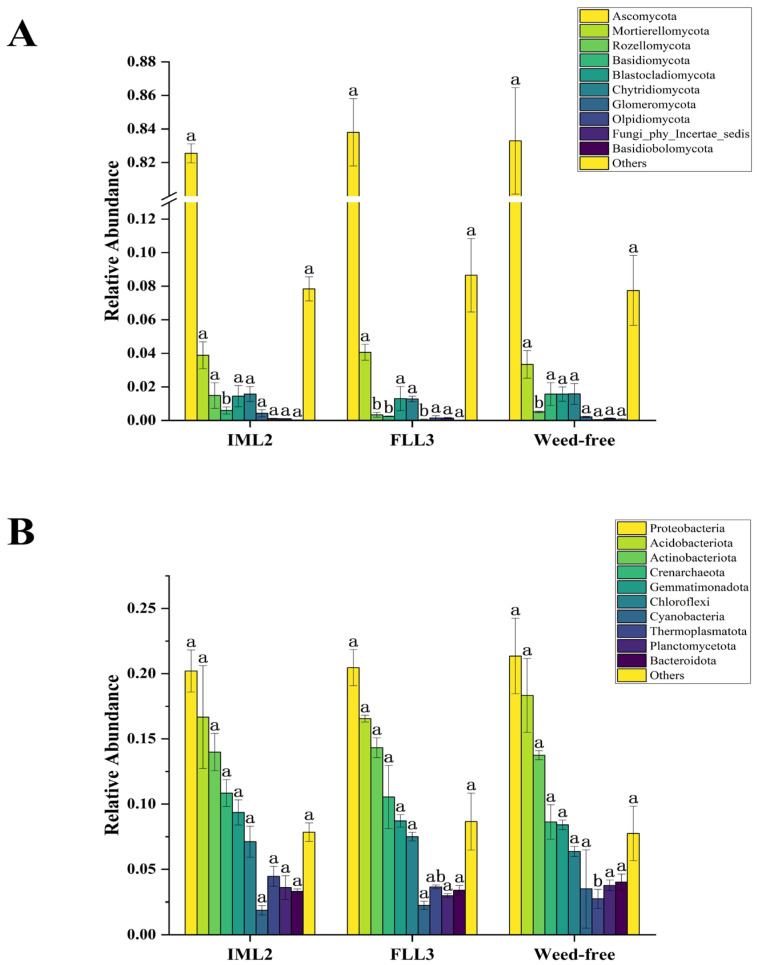
Column chart of relative abundances (%) of fungi (**A**) and bacteria (**B**) at the phylum level in the weed-free (control), IML2, and FLL3 treated soil samples. All treatments were measured with three replications. Different letters indicate significant differences between treatments (Duncan test at *p* < 0.05).

Microorganisms are important in soil ecosystems and agroecosystems. As with many agrochemicals, herbicides can also cause negative environmental impacts. Thus, a better understanding of the interactive responses of soil microbes toward herbicides is essential. We assessed soil microorganisms following pre-herbicide treatment at 30 DAS. Analysis of the relative abundances of fungal communities in each treatment (Figure 3A) revealed fungal taxa to include the phyla *Ascomycota*, *Mortierellomycota*, *Rozellomycota*, *Basidiomycota*, *Blastocladiomycota*, *Chytridiomycota*, *Glomeromycota*, *Olpidiomycota*, *Fungi-phy-Incertae-sedis*, and *Basidiobolomycota*. Of these, *Ascomycota* was universally most abundant, and that of *Mortierellomycota* was relatively stable. Compared with the weed-free (control) samples, there were no significant differences in soil fungal abundances between the IML2 and FLL3 treatments. Analysis of the relative abundances of bacterial communities across samples (Figure 3B) revealed the phyla *Bacteroidota*, *Planctomycetota*, *Thermoplasmatota*, *Cyanobacteria*, *Chloroflexi*, *Gemmatimonadota*, *Crenarchaeota*, *Actinobacteriota*, *Acidobacteriota*, and *Proteobacteria*. Among these, *Proteobacteria*, *Actinobacteriota*, and *Acidobacteriota* were highly abundant, whereas *Chloroflexi*, *Gemmatimonadota*, and *Crenarchaeota* occurred at moderate levels. As for fungal analyses, compared with the weed-free samples, there were no significant differences in the abundance of soil bacteria between IML2 and FLL3 treatments.

## 3. Discussion

*M. ruthenica* is an important forage legume in Northern China, especially in Inner Mongolia areas [24]. Due to overgrazing, only shrub and grass species were left in large parts of grasslands in Inner Mongolia. Ecological restoration of overgrazed grasslands requires forage species that can tolerate drought and cold winters. The candidate forage species can not only provide high forage yield and quality to the local beef and milk industry but also can adapt well to climatic conditions in this area. *M. ruthenica* is the best option, and it has been extensively used in this area due to its superior characteristics. However, its seed production is significantly challenged by weeds. Weeds pose many challenges in *M. ruthenica* seed production, including higher weeding costs and seed yield reduction, specifically at the time of its seedling establishment [25]. The use of selective herbicides is a more feasible option to control weeds on a large scale. However, there was no information about weed control by herbicides for *M. ruthenica*.

**Figure 4:**
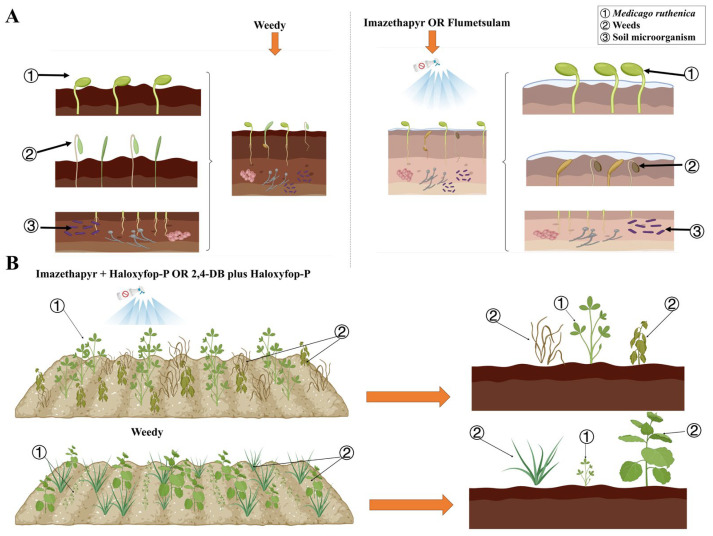
(**A**) Effects of pre-emergence herbicide application on *M. ruthenica*, weeds, and soil microorganisms. Compared with the control, applications of imazethapyr (1530 mL ha^−1^) and flumetsulam (120 mL ha^−1^) effectively reduce weed populations and do not negatively affect *M. ruthenica* crops and soil bacterial and fungal communities. (**B**) Effects of post-emergence herbicide application on *M. ruthenica* and weeds. Compared with the control, imazethapyr + haloxyfop-P (1800 + 600 mL ha^−1^) and 2,4-DB + haloxyfop-P (2250 + 600 mL ha^−1^) significantly reduce secondary weed infestation and promote *M. ruthenica* crop health (created in https://BioRender.com, accessed on 8 February 2025).

Pre-emergence herbicides are important for modern agricultural production to manage weeds [26]; determining the most appropriate herbicide to use requires an understanding of their effectiveness at controlling specific weeds, as well as possible crop injury and soil safety. We evaluate the effectiveness of weed control, crop injury, and soil safety of several pre-emergent herbicides in *M. ruthenica* (Table 1, Figure 1 and Figure 4A). Of them, acetochlor and pendimethalin are highly effective at controlling weeds, but they also significantly inhibit *M. ruthenica* plant height and dry matter accumulation. Imazethapyr and flumetsulam, particularly in IML2 and FLL3 treatments, also effectively suppressed weeds and exerted minimal impact on *M. ruthenica* plants. Additionally, we report that IML2 and FLL3 treatments do not significantly affect soil bacterial and fungal communities (Figure 3). Imazethapyr, when applied at 104 g ae ha^−1^ in alfalfa [22], and flumetsulam when applied at rates of 70 g ha^−1^ [27] and 0.05 to 0.14 kg a.i ha^−1^ [28] in soybean (*Glycine max*), significantly reduced weed emergence and legume biomass, which was consistent with our research. The efficiency of weed control and crop safety is affected by herbicide type and concentration [21], indicating that both factors influenced *M. ruthenica* growth and development.

Post-emergence herbicides play a crucial role in modern agriculture, particularly in the management of actively growing weeds [29]. Based on previous research, post-emergence herbicides are mainly used to control infestation weeds [30]. Because weed community composition and density vary after planting [31], no one herbicide may effectively control all weeds [32]. The tank mix of herbicides can effectively reduce the required dosage of individual herbicides and maintain or enhance weed control efficacy [33]. Haloxyfop-P effectively controls grasses [34]; thus, we mixed it with four post-emergence herbicides used to control broadleaf weeds. We report that imazapic + haloxyfop-P and oxyfluorfen + haloxyfop-P effectively control all weed species but significantly affect *M. ruthenica* plant height and dry matter. In contrast, the treatments with imazethapyr + haloxyfop-P and 2,4-DB + haloxyfop-P demonstrated relatively high effective control of all weeds without adversely affecting *M. ruthenica* plant height or dry matter (especially in IML3 + HA and 2,4L2 + HA treatments) (Table 2, Figure 2 and Figure 4B). Carvalho et al. reported that application of 2,4-D + haloxyfop-P (1005 g a.i ha^−1^ + 62.4 g a.i ha^−1^) effectively controlled approximately 50–60% of weeds [35]. However, we report that the weed-control efficiency of 2,4L2 + HA exceeds 86%. Combinations of haloxyfop-p-methyl at 135 g ha^−1^ + imazethapyr at 75 g ha^−1^ were recommended to control weeds in green gram (*Vigna radiata*) [36]. In our study, these combinations of post-emergence herbicides effectively control a wide range of weed species in *M. ruthenica*, which not only effectively manages a wider range of weed species but also contributes to the sustainability of agricultural practices by minimizing the dosage of herbicides.

Compared with other forage legume crops, weeds can outcompete *M. ruthenica* because it grows slowly as a seedling. Autumn sowing has been considered a method to conduct seed fields in the establishment year with reduced pressure from weeds. Although autumn sowing is a viable, effective strategy for weed control, it still has some issues: (1) Autumn sowing can negatively affect the hardiness and winter survivability of *M. ruthenica*. (2) Autumn sowing can lead to periods of idle land in spring and summer due to the absence of suitable crops with reproductive periods in northern China. (3) Autumn sowing reduces root and canopy growth and development before winter, resulting in a decreased number of stems in the following year and a subsequent decline in seed yield. Instead, chemical weed control has many advantages in *M. ruthenica* due to their flexible sowing, efficient weed control, and reduced costs associated with seed production in *M. ruthenica*. We demonstrate the potential of chemical control of weeds in *M. ruthenica*, which will enhance seed production and contribute to the longer-term viability of agricultural systems.

## 4. Materials and Methods

### 4.1. Location and Experimental Soil Properties

Experimental crops were planted in 2023 and 2024 at Saihan District, Hohhot City, Inner Mongolia Autonomous Region, China (111°53.40′ N, 40°43.70′ E). The soils had a pH of 8.1 and organic matter, nitrogen, phosphorus, and potassium contents of 1.29%, 0.084%, 10 ppm, and 260 ppm, respectively.

### 4.2. Weed Data Recording

Herbicides were applied to crops in accordance with the standards of the Ministry of Agriculture and Forestry, General Directorate of Agricultural Research and Policies (TAGEM) [12]. Weed species and densities were observed before experimentation. Within each experimental treatment, three replicate 1 m^2^ frames were randomly placed, and weed species and their numbers therein were recorded [12].

### 4.3. Plant Materials and Experimental Design

Plants (*M. ruthenica* (ND-3)) were sourced from the College of Life Sciences, Inner Mongolia University (Hohhot, Inner Mongolia Autonomous Region, China), and planted with a 15 cm spacing. The distance between treatments and each replicate was 1 m. For both years, experimental plots (5 × 4 m^2^) were arranged in a randomized complete block design with pre- and post-emergence herbicide treatments, each replicated thrice. Based on the previous research, pre- and post-emergence herbicides with potential for application were selected (Supplementary Table S2). To evaluate the selectivity and sensitivity of herbicides at controlling weeds in *M. ruthenica*, four pre-emergence and four post-emergence herbicide treatments were established. Pre-emergence herbicides imazethapyr (IML1, IML2, IML3), flumetsulam (FLL1, FLL2, FLL3), acetochlor (ACL1, ACL2, ACL3), and pendimethalin (PEL1, PEL2, PEL3) were applied 1 day after the sowing date of *M. ruthenica* seeds (DAS). Post-emergence herbicides of imazethapyr + haloxyfop-P (IML1 + HA, IML2 + HA, IML3 + HA), imazapic + haloxyfop-P (IPL1 + HA, IPL2 + HA, IPL3 + HA), 2,4-DB + haloxyfop-P (2,4L1 + HA, 2,4L2 + HA, 2,4L3 + HA), and oxyfluorfen + haloxyfop-P (OXL1 + HA, OXL2 + HA, OXL3 + HA) were applied 30 days after *M. ruthenica* seeds were sown. Herbicide details are summarized in Table 3 and Table 4.

### 4.4. Effects of Herbicides on Weeds

Weed population and dry matter data were measured at 30 DAS (pre) and 60 DAS (post). Weeds were sampled within three 1 m^2^ quadrants located on the diagonal in each plot. Aboveground weed biomass was harvested, identified, counted, and stored separately in paper bags, then oven-dried at 70 °C for 24 h to constant weight. The effect of herbicides on the total number of weeds within quadrats was determined using Equation (1) [12].

(1) $$Herbicide\;Percentage\;effect=\frac{\left(Number\;of\;Weeds\;in\;Control-Number\;of\;Weeds\;in\;Treatments\right)\;*\;100\%}{Number\;of\;Weeds\;in\;Control}$$

### 4.5. Effects of Herbicides on M. ruthenica Growth and Injury

Effects of herbicide treatment on *M. ruthenica* height and dry matter were determined at 30 and 60 DAS. At each harvest, *M. ruthenica* samples were collected from three 1 m^2^ quadrats; stems and leaves of each plant were removed and stored separately in paper bags, then oven-dried (70 °C for 24 h) to determine total dry matter. The condition of *M. ruthenica* plants in each treatment was assessed visually on a 4-point scale by comparing them with control plants, where 1 = very slight injury; 2 = slight injury; 3 = phytotoxic, and 4 = severely phytotoxic [23].

### 4.6. Effects of Pre-Herbicides on Soil Microbial Communities in M. ruthenica

Five rhizosphere soil samples (depth 0–20 cm) were randomly collected from each replicate pre-herbicide treatment plot 30 DAS. Plant tissues, debris, and gravel were cleaned from samples (passed through a 2 mm sieve). The five samples from each plot were mixed to form a mixed soil sample to produce three biological replicates for each treatment. After sample collection, the soils were stored at −20 °C for metagenomic analysis [37]. Significant differences between treatments were evaluated using Duncan’s multiple-range tests with a significance threshold of *p* < 0.05.

### 4.7. Statistical Analysis

Data were analyzed using SAS (Version 9.1) and RStudio (Version 3.4.1). Significant differences between treatments were evaluated using Duncan’s multiple-range tests with a significance threshold of *p* < 0.05. Figures were created using OriginPro 2021b.

## 5. Conclusions

Weed control is important for *M. ruthenica* cultivation. In this study, we first report the effects of various pre- and post-herbicides on weed control and crop safety in *M. ruthenica*. The results indicated that the most effective pre-emergence herbicides are imazethapyr (1530 mL ha^−1^) and flumetsulam (120 mL ha^−1^). Post-emergence herbicides that effectively control weeds and do not damage crops are imazethapyr + haloxyfop-P (1800 + 600 mL ha^−1^) and 2,4-DB + haloxyfop-P (2250 + 600 mL ha^−1^). The application of these herbicides can improve *M. ruthenica* seed production and the use of *M. ruthenica* in artificial grassland establishment and ecological restoration.

## Figures and Tables

**Table 1:** Effect of pre-emergence herbicide treatments on *M. ruthenica* crop injury.

Treatment	Timing	Crop Injury	2023		2024	
			Plant Height (cm)	Dry Matter (g/m^2^)	Plant Height (cm)	Dry Matter (g/m^2^)
IML1	PRE	3	19.17 ± 1.48 bc	3.42 ± 0.81 c	20.17 ± 1.59 ab	3.57 ± 0.65 d
IML2		1	20.83 ± 1.69 ab	6.15 ± 1.4 a	19.91 ± 2.02 ab	6.07 ± 1.24 a
IML3		2	18.33 ± 1.2 c	4.91 ± 0.82 b	16.33 ± 2.34 c	4.05 ± 0.52 c
FLL1	PRE	2	19.5 ± 3.18 ab	3.87 ± 0.64 c	20.5 ± 2.67 ab	3.94 ± 0.76 c
FLL2		2	17.67 ± 2.19 c	2.99 ± 1.17 d	18.75 ± 2.46 b	3.35 ± 1.36 d
FLL3		1	19.83 ± 2.77 ab	5.03 ± 1.28 ab	19.67 ± 1.97 ab	5.35 ± 0.84 ab
ACL1	PRE	4	6.50 ± 1.50 fg	0.82 ± 0.38 e	7.50 ± 1.36 e	1.25 ± 0.35 f
ACL2		4	6.00 ± 1.04 g	0.60 ± 0.24 ef	6.45 ± 1.22 e	0.63 ± 0.27 g
ACL3		4	5.17 ± 0.73 g	0.37 ± 0.19 f	6.09 ± 1.73 e	0.42 ± 0.13 g
PEL1	PRE	4	11.33 ± 0.67 d	2.11 ± 0.31 d	12.36 ± 1.67 d	2.31 ± 0.42 e
PEL2		4	7.83 ± 0.44 ef	1.05 ± 0.37 e	7.58 ± 1.47 e	1.00 ± 0.15 f
PEL3		4	8.83 ± 1.17 e	0.91 ± 0.07 e	6.22 ± 1.25 c	0.71 ± 0.07 fg
Weed-free			20.67 ± 0.88 ab	6.33 ± 0.34 a	19.73 ± 1.24 ab	5.97 ± 1.23 a
Weedy			22.53 ± 1.38 a	2.43 ± 0.06 d	21.42 ± 0.96 a	2.24 ± 0.10 e

Note: Pre-emergence herbicides imazethapyr (IM, IML1, IML2 and IML3 denote different concentrations), flumetsulam (FL, FLL1, FLL2, FLL3 denote different concentrations), acetochlor (AC, ACL1, ACL2, ACL3 denote different concentrations), and pendimethalin (PE, PEL1, PEL2, PEL3 denote different concentrations). All treatments were measured with three replications. Different letters indicate significant differences between treatments (Duncan test at *p* < 0.05).

**Table 2:** Effect of post-emergence herbicide treatments on *M. ruthenica* crop injury.

Treatment	Timing	Crop Injury	2023		2024	
			Plant Height (cm)	Dry Matter (g/m^2^)	Plant Height (cm)	Dry Matter (g/m^2^)
IML1 + HA	POST	1	20.00 ± 0.48 a	3.45 ± 0.04 ab	19.58 ± 0.78 bc	3.27 ± 0.23 b
IML2 + HA		1	18.00 ± 0.58 b	3.11 ± 0.50 b	19.34 ± 0.66 bc	3.65 ± 0.39 b
IML3 + HA		1	19.50 ± 0.87 ab	3.68 ± 0.09 ab	18.23 ± 0.57 c	4.35 ± 0.25 a
2,4L1 + HA	POST	1	20.23 ± 1.73 a	3.78 ± 0.05 ab	21.23 ± 1.56 a	3.49 ± 0.21 b
2,4L2 + HA		1	21.00 ± 2.89 a	4.16 ± 0.64 a	22.01 ± 1.95 ab	4.76 ± 0.37 a
2,4L3 + HA		2	15.00 ± 0.65 c	2.08 ± 0.45 c	14.00 ± 0.75 d	2.11 ± 0.22 c
IPL1 + HA	POST	4	13.00 ± 0.53 c	0.48 ± 0.27 f	14.00 ± 0.65 d	1.67 ± 0.12 c
IPL2 + HA		3	14.50 ± 2.60 c	1.66 ± 0.14 cd	13.26 ± 1.57 d	1.25 ± 0.14 d
IPL3 + HA		3	12.33 ± 2.17 c	1.01 ± 0.03 e	12.83 ± 1.95 d	1.12 ± 0.13 d
OXL1 + HA	POST	4	8.00 ± 2.00 d	0.86 ± 0.67 e	7.89 ± 1.37 e	0.98 ± 0.46 d
OXL2 + HA		4	6.33 ± 2.13 d	0.28 ± 0.18 f	7.24 ± 1.21 e	0.88 ± 0.34 de
OXL3 + HA		4	6.23 ± 0.17 d	0.36 ± 0.16 f	6.35 ± 0.87 e	0.76 ± 0.16 de
Weed-free			17.67 ± 1.20 b	3.79 ± 0.39 ab	18.67 ± 2.33 bc	4.33 ± 0.42 a
Weedy			18.47 ± 1.35 ab	1.62 ± 0.06 cd	20.48 ± 1.04 ab	1.88 ± 0.38 c

Note: Post-emergence herbicides of imazethapyr + haloxyfop-P (IM + HA, IML1 + HA, IML2 + HA, IML3 + HA denote different concentrations), imazapic + haloxyfop-P (IP + HA, IPL1 + HA, IPL2 + HA, IPL3 + HA denote different concentrations), 2,4-DB + haloxyfop-P (2,4 + HA, 2,4L1 + HA, 2,4L2 + HA, 2,4L3 + HA denote different concentrations), and oxyfluorfen + haloxyfop-P (OX + HA, OXL1 + HA, OXL2 + HA, OXL3 + HA denote different concentrations). All treatments were measured with three replications. Different letters indicate significant differences between treatments (Duncan test at *p* < 0.05).

**Table 3:** Characteristics of experimental pre-emergence herbicides.

Type	Treatments	Dosage Form	Formulation	Dose (mL ha^−1^)
PRE	Imazethapyr (5%)	AS	IML1	1275
PRE			IML2	1530
PRE			IML3	1800
PRE	Flumetsulam (80%)	SC	FLL1	60
PRE			FLL2	80
PRE			FLL3	120
PRE	Acetochlor (90%)	EC	ACL1	1050
PRE			ACL2	1200
PRE			ACL3	1350
PRE	Pendimethalin (33%)	EC	PEL1	1125
PRE			PEL2	1800
PRE			PEL3	2250
Weed-free	-	-	-	-
Weedy	-	-	-	-

Note: AS (aqueous solution), EC (emulsifiable concentrate), SC (suspension concentrate), and SL (soluble concentrate).

**Table 4:** Characteristics of experimental post-emergence herbicides.

Type	Treatments	Dosage Form	Formulation	Dose (mL ha^−1^)
POST	Imazethapyr (5%) + Haloxyfop-P (10.08%)	AS, EC	IML1 + HA	1200 + 600
POST			IML2 + HA	1500 + 600
POST			IML3 + HA	1800 + 600
POST	2,4-DB (30%) + Haloxyfop-P (10.08%)	SL, EC	2,4L1 + HA	1500 + 600
POST			2,4L2 + HA	2250 + 600
POST			2,4L3 + HA	3375 + 600
POST	Imazapyr (24%) + Haloxyfop-P (10.08%)	AS, EC	IPL1 + HA	225 + 600
POST			IPL2 + HA	300 + 600
POST			IPL3 + HA	375 + 600
POST	Oxyfluorfen (24%) + Haloxyfop-P (10.08%)	EC, EC	OXL1 + HA	375 + 600
POST			OXL2 + HA	450 + 600
POST			OXL3 + HA	600 + 600
Weed-free	-	-	-	-
Weedy	-	-	-	-

Note: AS (aqueous solution), EC (emulsifiable concentrate), SC (suspension concentrate), and SL (soluble concentrate).

## Data Availability

Data are contained within the article and Supplementary Materials.

## Supplementary Materials

The following supporting information can be downloaded at https://www.mdpi.com/article/10.3390/plants14060864/s1, Table S1: Dominant annual weeds and density (% m^2^); Table S2: Effect of pre- and post-emergence herbicides on weed control and crop injury.
