# Supplementary material for: Sustainable Weed Management: The Effects of Applying Pre- and Post-Emergence Herbicides to Medicago ruthenica
Source: Plants (Basel). 2025 Mar 10;14(6):864. doi: 10.3390/plants14060864 (PMC11945790; doi:10.3390/plants14060864)
Supplement: Supplementary file 1 [file plants-14-00864-s001.zip › Supplementary Table.pdf]

Table S1. Dominant annual weeds and density (%m^2).

		The Name of Weed	Density of Weeds(plants/m^2)	Relative Frequency of Weeds (%)
2023	Poaceae Barnhart	*Setaria viridis* (L.) P. Beauv.	24.70	9.25%
		Echinochloa crus-galli (L.) P. Beauv.	19.68	7.37%
		Chloris virgata Sw.	7.58	2.84%
	Chenopodiaceae	*Chenopodium album* L.	119.08	44.60%
	Amaranthaceae	*Amaranthus retroflexus* L.	72.70	27.23%
	Portulacaceae	*Portulaca oleracea* L.	6.73	2.52%
	Compositae	*Taraxacum mongolicu* Hand. -Mazz.	8.62	3.23%
	others	-	6.68	2.50%
			167.00	100%
2024	Poaceae Barnhart	*Setaria viridis* (L.) P. Beauv.	21.76	8.15%
		Echinochloa crus-galli (L.) P. Beauv.	17.06	6.39%
		Chloris virgata Sw.	9.75	3.65%
	Chenopodiaceae	*Chenopodium album* L.	108.51	40.64%
	Amaranthaceae	*Amaranthus retroflexus* L.	75.83	28.40%
	Portulacaceae	*Portulaca oleracea* L.	12.07	4.52%
	Compositae	*Taraxacum mongolicum* Hand. -Mazz.	14.93	5.59%
	others	-	8.30	3.11%
			183.00	100%

Tablet S2. Effect of pre- and post-emergence herbicides on weed control and crop injury.

Timing	Treatment	Crop Injury [a]	Weed control [b]
POST	2,4-DB (30%)	1	4
	Imazethapyr (5%)	1	5
	Metribuzin (48%)	4	3
	Bentazone (48%)	2	2
	Prometryn (50%)	4	5
	Clethodim (24%)	4	3
	Benazolin-ethyl (30%)	4	3
	Imazapyr (24%)	2	5
	Cloransulam (84)	3	2
	Oxyfluorfen (24%)	2	5
	Fomesafen (25%)	3	1
	Fluoroglycofen-ethyl (10%)	4	3
	Haloxyfop-P (10.08%)	1	4
PRE	Imazethapyr (5%)	1	5
	Imazamox (4%)	3	2
	Acetochlor (90%)	3	5
	Trifluralin (48%)	4	3
	Butralin (48%)	4	3
	Thifensulfuronmethy (75%)	4	1
	Flumioxazin (48%)	3	2
	Pendimethalin (33%)	3	4
	Clomazone (48%)	4	3
	S-Metolachlor (96%)	4	3
	Flumetsulam (80%)	1	4

Note: **a**, The condition of *M. ruthenica* plants in each treatment was assessed by visually on a 4-point scale by comparing them with control plants, where: 1 = very slight injury, 2 = slight injury, 3 = phytotoxic, and 4 = severely phytotoxic. **b**, the condition of weed control in each treatment was assessed visually on a 5-point scale by comparing them with control plants, where: 1 (0-20%), 2 (20-40%), 3 (40-60%), 4 (60-80%) and 5 (80-100%).
